# Fitness Characteristics of Jordanian Emergency Medical Technicians

**DOI:** 10.22114/ajem.v0i0.160

**Published:** 2019-07-01

**Authors:** Hani Najeh Mansour Al-Yousef, Wael Awada, Evangelia Michailidou

**Affiliations:** 1. Al-Ghad International College for Applied Medical Science, Riyadh, Saudi Arabia; 2. EMS Department, Al-Ghad International Health Sciences Colleges, Riyadh, Saudi Arabia; 3. Department of Anesthesiology and Intensive Care and Pain Management, Cairo University, Cairo, Egypt; 4. Intensive Medicine Department, Hippokration General Hospital, Thessaloniki, Greece; 5. Department of Disaster Medicine and Health Crisis Management, University of Athens Medical School, Athens, Greece

**Keywords:** Emergency Medical Technicians, Jordan, Habits, Health, Overweight, Physical Fitness

## Abstract

**Introduction:**

Emergency medical technicians (EMTs) should be always prepared to deal with the stressful condition of treating patients with serious physical and emotional injuries. Given that EMTs consider safety the first priority, they must pay adequate attention to their own physical well-being and fitness to practice.

**Objective:**

The present study was conducted to analyze the fitness of Jordanian EMTs.

**Method:**

The present prospective study was conducted to evaluate the well-being of Jordanian paramedics. The survey was designed using Google forms, which were completed by the participants. The data collection tools comprised an already-designed checklist, including items such as age, gender as well as height and weight, which are used for calculating body mass index (BMI). In addition, the presence of chronic diseases such as hypertension, diabetes mellitus, renal failure and cardiorespiratory diseases as well as the history of surgeries and disabilities were investigated. The participants were also asked about their smoking status and other health-related habits.

**Results:**

Out of 115 surveys conducted, 7 were discarded owing to loss of information or making completion mistakes. Out of the remaining 108 respondents, 82 (75.9%) were male and below 10% were over the age of 30 years. BMI was over 25 in 40.7% of the respondents, and only 4 (3.7%) had chronic diseases. Moreover, 46 (42.6%) respondents were smokers, and only 30 (27.8%) performed regular exercise.

**Conclusion:**

The present findings suggest health problems in a small percentage of the EMTs, potentially due to the appropriate support provided by the employers or university authorities in this regard. The major health problem was overweight and unhealthy lifestyle, including smoking and not doing regular exercise.

## Introduction

As the healthcare professionals trained to provide emergency out-of-hospital assessments and care for people who are injured or sick, Emergency Medical Technicians (EMTs) should be always prepared to deal with the stressful condition of treating patients with serious physical and emotional injuries. Given that EMTs consider safety the first priority, they must pay adequate attention to their own physical well-being and fitness to practice (1). The state of physical well-being is not limited to the absence of diseases, and encompasses the selection of a proper lifestyle required for ensuring health, preventing diseases, and enjoying a balanced state of body, mind and spirit (2). From the standpoint of EMTs, physical well-being includes three major components, namely physical fitness, healthy habits and adequate sleep. Physical fitness is required for safely lifting and moving patients as a critical part of their job without harming themselves. Healthy habits refer to performing regular exercise, having good nutrition to build their body, and avoiding bad habits such as smoking, drinking and drug use. Adequate sleep is also required given that working shifts can conflict with their body’s natural rhythm and create physical, mental and social problems. EMTs can be exposed to different infectious diseases such as hepatitis B and acquired immune deficiency syndrome (AIDS) or musculoskeletal injuries caused by assessing and transporting mentally-unstable or combative patients. Maintaining high levels of physical fitness is therefore a key requirement in EMTs for doing their job safely (3). The present prospective study was therefore conducted to evaluate well-being in Jordanian paramedics.

## Methods

The present prospective study was conducted in the late 2018 to assess the well-being of Jordanian paramedics and expand knowledge in this regard. The present research supports the values required for collaborative work, including mutual respect and fairness. The present study was not financially supported by the government. The principles of conducting research on human or animal subjects were also respected. The authors declare no conflicts of interest, and support important social and moral values such as the principle of causing no harm to others. The survey was designed using Google forms, which were completed by the Jordanian paramedics of both genders, who worked for the Jordanian civilian defense sector and emergency departments of different hospitals. The data collection tools comprised an already-designed checklist, including age, gender and body mass index (BMI). In addition, the presence of chronic diseases such as hypertension, diabetes mellitus, renal failure and cardiorespiratory diseases as well as the history of surgeries and disabilities were investigated. The participants were also asked about their smoking status and other health-associated habits. All the statistical analyses were performed in IBM SPSS Statistics for Windows, version 19.0 (IBM Corp., Armonk, N.Y., USA).

## Results

Out of the 115 surveys conducted, 7 were discarded due to loss of information or making completion mistakes. Out of the remaining 108 respondents, 82 (75.9%) were male and below 10% were over the age of 30 years. Table 1 presents the characteristics of the participating EMTs.

**Table 1:** Descriptive characteristics of the participating EMTs

**Variable**	**Frequency (%)**
**Gender**	
Male	82 (75.9)
Female	26 (24.1)
**Age (year)**	
18–25	54 (50.0)
26–30	44 (40.7)
>30	10 (9.3)
**BMI**	
<18.5	62 (57.4)
18.5–24.9	44 (40.7)
>25	2 (1.9)
**History of chronic diseases**	
Diabetes mellitus and hypertension	1 (0.9)
Hypertension and herniated disc	1 (0.9)
Asthma and herniated disc	1 (0.9)
Heart failure	1 (0.9)
**History of surgeries**	
Appendicitis	6 (5.6)
Herniation	2 (1.9)
Gunshot injury	1 (0.9)
Varicose veins	1 (0.9)
Bone fracture	1 (0.9)
Tonsillectomy	1 (0.9)
Ranula	1 (0.9)
**Disability**	
Vision problems	4 (3.7)
Glaucoma	1 (0.9)
Simple hearing loss	1 (0.9)
**Habit**	
Smoking	46 (42.6)
Regular sport program	30 (27.8)

Although a normal BMI was observed in over half of the respondents, 40.7% were overweight with a BMI>25. Chronic diseases were observed in only 4 (3.7%) respondents, and different abnormalities in only 6 (5.6%), while 13 (12%) had history of surgeries. Forty-six (42.6%) respondents were also smokers, and only 30 (27.8%) performed regular exercise Moreover, six (5.6%) participants reported not to have received enough training on patient lifting and moving skills. Furthermore, fifty-four (50%) of the respondents had difficulties lifting and moving patients, and 75 (69.4%) believed working as an EMT would negatively affect their health in the future.

## Discussion

EMTs’ wellness is obviously affected by different factors such as BMI, which was found to suggest overweight in about 40.7% of the respondents. This high and unacceptable percentage of overweight subjects is mainly associated with adopting an unhealthy lifestyle, which manifested itself as a low percentage (27.8%) of the EMTs performing regular exercise, and the 42.6% of the participants being smokers. Chronic diseases were observed in only 3.7% of the subjects, all of whom were below the age of 45 years. Although the percentage of EMTs exposed to surgery was 12%, most of these surgeries, including appendicitis, ranula, herniation, varicose veins and tonsillectomy, were uncomplicated to be performed in the young. Only two (below 2%) of the EMTs were exposed to surgeries that could potentially affect their work. Hereditary problems, including vision and hearing disorders, were the only type of abnormality, which was observed in 5.6% of the EMTs. A total of 94.4% of the EMTs reported receiving enough training on patient lifting and moving skills, which is a normal figure given that receiving immense training on these skills is essential for EMTs; nevertheless, 50% of them had difficulties lifting and moving patients, which can be explained by several factors such as patient weight and the health status of the EMTs. Furthermore, 24.1% of the sample were female with natural difficulties doing hard tasks. The majority of the EMTs had a positive attitude towards providing health services for patients, as about 70% believed they might face health problems in the future. According to the results obtained, EMTs are recommended to be encouraged to take up a healthier lifestyle, and especially perform regular exercise and avoid smoking and alcohol consumption. The overweight EMTs are also recommended to go on a diet. In addition, working hours are recommended to be decreased and an annual health assessment to be performed in all the employees.

## Conclusion

The low percentage of the EMTs with health problems suggests the appropriate support provided by the employers or university authorities in this regard. However, the major health problems observed included overweight and unhealthy habits such as smoking and not having regular exercise.
